# Collaboration and Communication in Care at the Nursing Home: The Next of Kin’s Experiences of Participation Following Educational Intervention for Staff

**DOI:** 10.3390/nursrep15070255

**Published:** 2025-07-14

**Authors:** Helene Åvik Persson, Birgitta Wallerstedt, Åsa Alftberg, Anna Sandgren, Gerd Ahlström

**Affiliations:** 1 Department of Health Sciences, Faculty of Medicine, Lund University, SE-221 00 Lund, Sweden; gerd.ahlstrom@med.lu.se; 2 Center for Collaborative Palliative Care, Department of Health and Caring Sciences, Faculty of Health and Life Sciences, Linnaeus University, SE-351 95 Växjö, Sweden; b.wallerstedt@telia.com (B.W.); anna.sandgren@lnu.se (A.S.); 3 Department of Social Work, Faculty of Health and Society, Malmö University, SE-205 06 Malmö, Sweden; asa.alftberg@mau.se

**Keywords:** care home, elderly care, family member, interview study, involvement, nursing home, palliative care, relatives, residential facilities

## Abstract

**Background**: After an older person moves into a nursing home, the next of kin often continues to participate in the care provided there. This participation in care may contribute valuable knowledge of the preferences and wishes of the older person, thereby helping nursing staff deliver personalised care. **Objectives**: The aim of this study was to explore how next of kin experience their participation in the care of older persons residing in nursing homes after educating nursing staff about participation in palliative care. **Methods**: This follow-up study used a qualitative design based on semi-structured interviews with 37 next of kin. A thematic analysis was applied. **Results**: Participation of the next of kin involved active communication and collaboration with nursing staff, expressed in three themes: striving to achieve co-created care, navigating involvement through presence, and building commitment through communication and information. The dual role of being an emotionally close next of kin and a participant in the relative’s care was challenging and became increasingly burdensome and often overwhelming when the older person’s health deteriorated. **Conclusions**: This study reveals the need to develop and implement a policy for the participation of next of kin in the care of older people living in nursing homes. In addition, support groups can increase well-being through dialogue with other next of kin, thereby alleviating emotional strain. Increased implementation of life stories and the use of digital communication would keep the next of kin informed about the older person’s condition, especially when they cannot be present in person. Life story is a valuable tool for person-centred care and strengthens the relationships between the next of kin, the older person, and the nursing staff.

## 1. Introduction

A next of kin’s care for an older person is described as a never-ending responsibility that is both voluntary and compulsory [1]. The most common and rapidly growing need for care is among older people, largely due to an ageing population. As a result of increasing life expectancy, the proportion of people aged 80 and over is expected to increase by more than 50 percent by 2030. This will place greater demands on both the next of kin and society [2,3]. Home care is the preferred model, based on the principle of “ageing in place” [4]. Moving into a nursing home usually occurs when the older person is too ill or frail to continue living at home. Swedish legislation [5], guarantee support with the ambition of preventive and evidenced-based social service. Care in nursing home are provided when an older person’s needs are assessed as being extensive and requiring a high number of daily interventions that exceed the capacity of the municipality’s home care service. This means that the older adult requires support with multiple daily activities, such as moving around, getting in and out of bed, outdoor and social activities, cooking, personal hygiene, and grooming. A two-tiered care structure in nursing homes means that when an older person requires healthcare, for example, the administration of medication, it is regulated by the Health and Medical Services Act [6].

The nursing home is a common place for care until death for a significant number of frail older people with progressive illnesses and anticipated deterioration [7,8,9]. Frail older people living in nursing homes often have multiple chronic diseases and dementia, with complex requirements for person-centred and palliative care [7,8,10,11]. Next of kin are devoted companions of the older person in the last phase of life, participating in the transition towards death [12]. This process has emotional, physical, and social impacts on the next of kin, so it is crucial for staff to understand their situation. One of the four core pillars of palliative care is supporting the next of kin. This means that the next of kin should feel included in the care process and receive information and support tailored to their needs and specific circumstances [13]. Although the next of kin may be considered ‘part of the team’ in providing older people’s care and service, nursing homes often lack structures to facilitate their participation and engagement in care [14,15,16]. Participation (also referred to as involvement) is vital for the next of kin’s experiences of high-quality care in the nursing home [17,18,19]. However, it can be challenging to implement in practice [20].

Several studies show that the next of kin remain involved in the lives of their loved ones after placement in the nursing home and constitute an intertwined unit [9,20,21]. The next of kin’s participation in care includes visiting, providing socio-emotional care, advocacy, and personal care [20,22]. An emotional dilemma for the next of kin has been expressed in terms of a balancing act between maintaining one’s own responsibility for the care while, at the same time, leaving the responsibility of the care to the staff [23]. Next of kin play an important role in the lives of older person, participating in, and sometimes even taking over, the decision-making process when their health is at stake. Therefore, it is also important to consider the next of kin’s satisfaction with the care offered at nursing homes [24]. Support from staff in terms of information, communication, and understanding facilitates the next of kin’s own decision and willingness to participate in the care [25] and alleviates their feelings of guilt [26]. The expertise of next of kin must be acknowledged, and staff need to see them as resources with knowledge of the older person’s life story, i.e., past interests, activities, personality, and family relationships [22,27]. The ambiguity that arises when the next of kin and nursing staff lack a shared understanding of what positive participation might be makes it difficult for the next of kin to participate in ways that are meaningful and rewarding [27]. Next of kin who have the opportunity to choose the level of involvement based on their life situation participate in the care more meaningfully, which has benefits for both the next of kin and the older relative [28,29]. Making the next of kin’s experiences visible may help care staff to respond adequately and provide the necessary support.

Caring for dying older people and their families requires integration of palliative care into current care work; however, little is known about the different ways in which palliative care is being implemented in the nursing home setting internationally [30]. A systematic review identified only two RCTs and one controlled before-and-after study of multi-component palliative care interventions in the USA [31]. These interventions were evaluated in different ways; the populations included and the outcome measurements differed. Knowledge of how to improve palliative care in these settings remains scarce even in Europe. An EU-funded project, Palliative Care for Older People (PACE), implemented a train-the-trainer programme for staff for one year. The programme was evaluated among older persons and staff in a cluster-randomised controlled trial involving Belgium, the Netherlands, England, Finland, Poland, and Italy. The outcomes of the evaluation revealed a large difference in how palliative care was implemented across the six countries [32,33,34]. The Implementation of the Knowledge-Based Palliative Care in Nursing Homes (KUPA) project was developed in Sweden to deliver an educational intervention to staff in two counties and consisted of five seminars and self-studies on knowledge-based, general palliative care for 6 months. A structured curriculum designed for nursing home staff was used [35]. The published study protocol describes the multi-measurements conducted on older persons, their next of kin, staff, and managers using qualitative and quantitative methods [36]. The design of qualitative and quantitative data regarding next of kin’s participation was based on the fact that there is no consensus in the literature on the definition of participation [37] and no instrument has been identified for assessing the participation of next of kin in nursing home care. This sub-study explored how the next of kin experience their participation in the care of older persons residing in nursing homes after educating nursing staff about participation in palliative care.

## 2. Methods

### 2.1. Study Design

This follow-up study employed an explorative qualitative design based on semi-structured interviews with the next of kin. A qualitative reflexive thematic analysis based on the method of Braun and Clarke [38] was applied.

### 2.2. Nursing Home Setting

The Social Services Act [39] is a piece of legislation for older people in the community who need care and support with activities of daily life. The Swedish care model is publicly financed and available to all citizens in need [1,2,39]. Sweden has, for the last four decades, prioritised providing care to older persons in their own homes for as long as possible [4,40,41] using a two-tiered care structure: social services and elderly care offered by the municipality, and healthcare by the county council. Swedish eldercare consists of care in the home (help getting dressed, showering, cleaning, meals, etc.) and care in the nursing home (formally known as ‘special housing for the elderly’); the latter is provided on-site 24 h a day, 7 days a week, including care by on-site or off-site registered nurses and off-site general practitioners. The requirement for receiving an apartment in a nursing home with everyday care is high; only those who need extensive care and service are entitled to a place [42]. A social worker in the municipality is responsible for assessing and making decisions in line with the Social Services Act [39] and ensuring that the individual’s need is significant enough to warrant relocation to a nursing home. Nursing homes in Sweden aim to create a homelike environment and provide small, private apartments with their own lease. These apartments typically consist of one room, a small kitchen area, and an accessible bathroom. The apartments are often clustered together in the same building, with shared spaces designed to foster a sense of community. A variety of group activities are available in nursing homes, encouraging social interaction and supporting the older person’s overall well-being [39,42].

Staff members are present around the clock to provide necessary continuous care for the residents and are employed by the municipality (referred to in this study as nursing staff). Many nursing staff are trained as assistant nurses; some may have limited educational backgrounds with no specific training in gerontology or geriatrics [43]. There are also registered nurses, those working in rehabilitation as occupational therapists, and physiotherapists. Unit managers often have a background in social work, with at least a bachelor’s degree. The Swedish National Board of Health and Welfare set the goal that everyone should have access to knowledge-based palliative care [13,44], and the Social Services Act has recently set the same goal for social services [39]. However, knowledge of palliative care among nursing staff is varies in extent [45,46].

In Sweden, it is common for the next of kin to participate in the care of their older relatives, even if they have no formal responsibility [42]. Since 2009, municipalities have been obligated to offer support to next of kin who provide care or support to someone close to them. Support is characterised by individualisation, flexibility, and quality [47].

### 2.3. Research Setting

This study is part of the educational intervention for nursing staff in the KUPA project. Knowledge-based palliative care in this project means research- and practice-based knowledge [36]. This project used a non-randomised experimental design, with intervention and control groups, as well as pre- and post-assessments [36,48]. The participating nursing homes consisted of a mix of both larger (≥100 older persons) and smaller (<25 older persons) nursing homes in both urban and rural settings. The next of kin were selected from the nursing homes in the two counties in southern Sweden [36]. They were selected based on the size of each nursing home, ranging from four in the largest nursing home to one in the smallest nursing home.

Three previous studies about the next of kin’s participation are published within the KUPA project. The first quantitative study consisted of a psychometric evaluation of the newly developed self-report questionnaire called Next of Kin Participation in Care (NoK-PiC) with 260 respondents [37]. This was additionally evaluated (n = 203) using traditional within-group and modern within-individual analytical approaches following the intervention and comparing the outcome between the intervention and control groups [49]. The scale was found to be sensitive in measuring between-group comparisons and showed an improvement in participation in the intervention group compared with the control group on both Communication and Trust (CaT) and Collaboration in Care (CiC) subscales. The qualitative interview study before the educational intervention (n = 40) revealed an overarching theme of a balancing act between wanting to maintain and wanting to relinquish their responsibility for the old person’s care. The previous results are compared with the results in the current study in the Discussion section.

### 2.4. Educational Intervention

The educational intervention consisted of five seminars, 3 h each, including refreshments, based on existing knowledge about palliative care [36]. Details of the development, pre-testing, and implementation of the intervention are described in more detail in the published study protocol. A training booklet was designed to cover the five topics of the seminars (Table 1), including lectures, videos, and a reference list for self-studies by the nursing staff. The booklet also included assignments for individual and group preparations of each seminar, as well as tasks to be completed between each seminar [35,36]. Each seminar group consisted of 8–10 nursing staff who would bring ‘cases’ from their daily work to the seminar discussions, and in this way, the content was adapted to the context of the respective nursing homes. In parallel, the content and discussions from the seminars would be transmitted by the participants to all colleagues in their workplace to initiate improvements in nursing care.

### 2.5. Participants in the Follow-Up Study

The inclusion criterion for this study was that the next of kin must have participated in the previous interview, which took place nearly one year prior [23]. The inclusion criteria in the first interview before the educational intervention were as follows: (1) be a next of kin to an older person living in a nursing home, (2) be well-known by nursing staff, i.e., have frequently visited their older person or have frequent phone contact, and (3) be able to speak and understand Swedish [23]. The participants in this study were recruited from 20 nursing homes in each of the two counties (16.4% of Sweden’s population). Among the 40 next of kin invited from the previous interview [23], 3 chose not to participate in this follow-up interview. The reasons for not participating were their own ill health, the death of the older person, and no specified reason. The thirty-seven next of kin who participated were between 50 and 89 years old; the majority were women, married, and a child of the old person, had a college education, worked full-time, and made recurrent visits to the nursing home (Table 2). One next of kin did not provide any characteristics about themselves.

### 2.6. Data Collection in the Follow-Up Study

Initially, the researchers asked the participants in the previous interview [23] via phone whether they wished to participate in the present research. Information was given again about the study, and, for those who wanted to continue, a time and place for an interview were determined based on the next of kin’s wishes. Before the interview, oral and written information about the study was provided, and written consent was obtained from each participant. The interviews took place at a location chosen by the next of kin: in a conference room, at the nursing home, or in the next of kin’s private home.

The interviewers were registered nurses with extensive experience working in older care, as well as conducting research interviews. They conducted the interviews according to a pre-defined semi-structured interview guide. The participants were asked the same questions as before the educational intervention [23] regarding their participation in care. The opening question, “*In what ways do you participate in the care of your relative at the nursing home*?” was followed by two questions: “*How would you like to participate*?” and *“Have you experienced any obstacles to participate*?”. The number of in-depth questions depended on the richness of the participants’ initial answers, and they were expressed as “*Could you tell me more about your experiences*?”, “*What did you think in that situation*?”, and “*What did you do in that situation*?”. A supplementary question was added when only a few next of kin spontaneously mentioned specific palliative care: “*Have you provided the possibility to communicate your thoughts about palliative care concerning your relative*?”.

These follow-up interviews, conducted nine to eleven months after the initial ones, March to May 2018, ranged from 25 to 90 min (median: 42 min; mean: 46 min). All the interviews were digitally recorded and transcribed verbatim.

### 2.7. Data Analysis

The interviews were analysed using Braun and Clarke’s [38] reflexive thematic analysis process. The step-by-step approach described below was applied, and NVIVO 11 [50] was used to code the data. The analyses were conducted independently by the first and second authors (HÅP and BW, respectively).

First, data familiarisation was achieved by listening to the recorded interviews and repeatedly reading all the transcripts, allowing for initial reflections and interpretations. Next, relevant data were systematically organised into initial codes by open coding and collecting the data relevant to each code. Codes were created by assigning distinct labels to specific sections of the text. These initial codes were then grouped into potential sub-themes, which involved combining codes into themes that depict the data. Thereafter, the sub-themes were reviewed by comparing them against the coded extracts and the entire dataset to ensure coherence and thematic validity. Further, a few sub-themes were reworded to better reflect their meaning, and later, all were organised into themes. Themes were reviewed and clearly defined, enabling the consolidation of similar themes and ensuring clarity in the final selection. All authors reviewed the themes and sub-themes and discussed the analysis until a consensus was reached regarding the results.

Finally, a detailed description of the results was written. To enhance robustness, the results are supported by quotes that illustrate the findings. Examples of the analytical procedure are given in Table 3.

## 3. Results

The thematic analysis yielded three themes and nine sub-themes (Table 4).

### 3.1. Striving to Achieve Co-Created Care

Next of kin highlighted the importance of good relationships with nursing staff, which they found needed a balance of their different roles and setting boundaries. This, in turn, strengthens their relationship with the nursing staff, thereby enabling both family involvement and trust in staff expertise. A crucial goal that emerged from their interaction was building and maintaining trust, which lays the foundation for respectful and safe care for their older relatives. They also expressed organisational obstacles such as the high turnover of staff and a lack of skills.

#### 3.1.1. Balancing Dual Roles and Boundaries

The next of kin described the challenge of managing dual roles as both family members and informal carers, where their involvement in care required constant adjustment. As the older person’s health deteriorated, these responsibilities became even more demanding, requiring them to continuously reassess and adapt to new expectations and needs, an often-overwhelming process. This balancing process made it more challenging to cope with the dual roles of both the family member and informal carer when they bore sole responsibility for the older person.

The roles of next of kin inherently involved emotional conflicts, as they strove to provide the best possible care for their loved ones while also seeking the freedom to maintain their own lives. Without clear boundaries or support, the demands of care became overwhelming, highlighting the emotional and physical strain of navigating these intertwined roles. The feeling of loneliness in the role of next of kin was a considerable source of stress, as the heavy burden placed on their shoulders often made them feel inadequate. These feelings reappeared despite receiving support from other people around them. Being alone in the caring role, with no siblings or children to share the responsibility with, increased the feelings of inadequacy and loneliness.

*It probably would have been a lot easier if I had siblings because then I could have dealt with it in a different way because then I know that there’s still someone close to me but because I’m an only child I don’t have anyone here. So it’s just me and my mum, so it didn’t feel good*.

(Son)

Balancing the role of next of kin also had an impact on their physical and mental health. Some next of kin reported a good quality of life when they knew their loved one was well cared for, while others struggled with mental health problems as the older person’s condition deteriorated.

*No, it’s not possible, it’s really hard, terribly hard because I feel so bad that he’s not feeling well*.

(Daughter)

#### 3.1.2. Strengthening the Relationship

The relationship between the next of kin and nursing staff is crucial in the care of older persons, where mutual understanding and collaboration can strengthen their bond. They found the nursing staff very friendly and helpful, where confirmation, respect, and knowledge about each other facilitated the relationship. However, next of kin expressed an ambiguity in their feelings regarding the relationship with the nursing staff. On the one hand, they emphasised the desire and need for involvement in the care of the older person, but on the other hand, they recognised the challenges and uncertainty in carrying out the unclear role of the next of kin in collaboration with the expertise of nursing staff. Their experiences of challenges were related to organisational changes, such as nursing staff reductions, newly employed staff, and a lack of skilled personnel, which negatively impacted the care provided to the older person and made it more difficult to meet the older person’s individual needs and maintain a proper relationship. The next of kin found themselves acting as intermediaries between the older person and nursing staff, such as with dentists or external specialists. They wished for greater support from nursing staff in ensuring that necessary appointments were scheduled and followed through. A more proactive approach of nursing staff in coordinating care was seen as essential to maintaining a good relationship with the next of kin.

*I have no feeling for it anyway, … I don’t know. But there’s also the thing about duty: ‘Ah, I’m not here when the dentist comes’, one of the nursing staff said, no, there’s a bit of a gap…*.

(Daughter)

A key motivator for the next of kin’s active participation in care was a profound sense of gratitude towards their parents, which was a recurring theme in the next of kin’s experiences. Some of the next of kin reviewed their involvement as a way of giving back to their parents and honouring them during their final stage of life. Their involvement was something that the nursing staff mostly appreciated and supported.

*It’s natural, you feel that you… want to give something back, you want to show that you appreciate what they’ve done for me and that I understand that they’ve made a lot of sacrifices to help… all their children*.

(Daughter)

For spouses, the relationship with the nursing staff and the nursing home continued even after the passing of their loved ones. Several continued to visit the facility; these visits reflect the positive care relationship with the loved one that extends beyond the life of the older person. Maintaining contact with nursing staff was a way of showing gratitude for the care provided, highlighting the emotional connections that had developed within the care environment.

#### 3.1.3. Building Trust

Several key prerequisites for participation were highlighted by the next of kin as building trust, such as continuity of care, respect, attentiveness, accessibility, and support. Many next of kin emphasised the importance of having the same nursing staff caring for the older person, as this enabled the development of trust and gave them confidence in the care provided. One example of this was having a designated contact person to help establish a relationship. For some next of kin, it was important to trust the nursing staff, which meant allowing them to carry out their work without interference or questioning.

*I don’t want to get involved, I worked in a pharmacy for 15 years so I know a lot about medicines, but I would never get the idea of going into their… job and making comments and stuff like that, so I fully trust them, that it’s taken care of*.

(Daughter)

However, some next of kin experienced a greater sense of responsibility, which stemmed from a lack of trust in the care system or the perception that time constraints for nursing staff explained the poor quality of care. The widespread perception among next of kin is that care is constrained by a shortage of nursing staff, affecting both the quality of treatment and the overall well-being of older persons. Some next of kin described feeling the need to direct nursing staff in their work to ensure that appropriate care is provided.

*It’s absolutely incredible! The other day I came here at half past nine, when I came in there. Mum was sitting in her nightgown, at the table there, ‘Well, haven’t you got your clothes on yet?’, ‘No’ she said ‘They have so much to do…. I had breakfast before I got dressed because I had to shower’. ‘Well, it was good that you got breakfast then’. I was still there, it was nearly eleven before anyone had time to come in and help her shower so she could get dressed. Well, it was lucky she had had breakfast, because then at half past eleven they must go to lunch*.

(Daughter)

### 3.2. Navigating Involvement Through Presence

The role of the next of kin in the care of older persons living in nursing homes is multifaceted, encompassing both practical assistance and emotional presence. This involvement ranges from everyday visits, such as helping with daily and personal needs, to active advocacy for quality care and older persons’ rights. The next of kin’s experiences shed light on the responsibilities they navigate through their commitment to presence and support.

#### 3.2.1. Visiting the Nursing Home

The next of kin’s willingness to make visits was clear, and they prioritised these when opportunities arose.

*I always used to joke in the morning when I arrived (sounds happy), because you get to know the nursing staff quite well here, so then I always say, Now ‘Sister Allan’ is coming to report for duty today. There were good relations with the nursing staff and me and there still are*.

(Son)

Despite their deep care and commitment, some next of kin struggled with feelings of grief and helplessness as the older person’s personality and recognition faded. This emotional burden sometimes led to a sense of detachment or reluctance to visit as frequently as before.

*But it’s hard sometimes when you leave here, you feel like you’re completely exhausted*.

(Daughter)

For other next of kin, the visits were even more important when the health of their loved one was deteriorating, as their time with the older person seemed to be limited. Those next of kin who experienced bad conscience felt that they had contributed to the old person moving to the nursing home. Because of this, the frequency of visits was important, and the next of kin stated that they wanted to visit more frequently but did not always have the opportunity.

#### 3.2.2. A Helping Hand

Being regarded as a resource to the old person and the nursing staff was a very positive experience for the next of kin and increased the energy and willingness to participate in care. While there was a willingness to offer a helping hand in the care process, other next of kin emphasised the importance of being on equal terms and not being taken for granted by the nursing staff. It was important that their involvement was based on mutual agreement, ensuring that their participation was recognised as voluntary and appreciated rather than expected. Some next of kin stressed the importance of participation always being a choice, not an obligation. For some next of kin, lending a helping hand at the nursing home had become a daily routine, creating a sense of meaning.

*They (the nursing staff) know when I come up here in the evening, they know that I’m usually here at a quarter after five, when their dinner is ready and it’s just standing there*.

(Husband)

Several of the next of kin felt that they had to take on significant responsibility for everyday tasks, such as feeding and laundry. For some, providing practical support felt like a natural part of caregiving, whereas others found the responsibility overwhelming, particularly when adequate support from nursing staff was lacking. However, several next of kin also found that nursing staff did not want to involve them in care.

*I would like them (the nursing staff) to be more involved. I don’t want to point fingers because they probably have a lot to do that I don’t know about. They could ask next of kin for help with certain things, because I am not afraid to lend a hand. My mum is so lucid that she can do certain things on her own, on certain days. Other days she’s dizzy and doesn’t know how to get dressed, she has different days*.

(Daughter)

On the other hand, the next of kin expressed relief at being able to hand over practical responsibilities to the nursing staff. By not having to focus on daily tasks, they could instead concentrate on being emotionally present during visits, which helped strengthen their relationship with the older person.

*I think it feels really nice for me to come here and socialise. She has money in her safe, so if any consumable items are missing, they (the nursing staff) can go and fix it*.

(Daughter)

#### 3.2.3. Advocating for Quality and Rights

The next of kin described having a central role in ensuring that the older persons received safe and dignified care in the nursing home. They saw themselves as the older person’s voice through dialogue with nursing staff, and they ensured that their views and wishes were taken into consideration. This involved being present and engaged but also having the courage to advocate for quality and rights within the nursing home. However, next of kin expressed a fear of being perceived as difficult, which made them hesitant to voice concerns openly. Thus, this dissatisfaction was sometimes implied rather than explicitly stated, making it challenging for the next of kin to drive change in the care process.

*Because she was very… I recognise it because she’s had so many urinary tract infections which I pointed out. I shouldn’t have done that because it was none of my business. So, they changed her medication again and haven’t told me. But I’ve heard that she’s still quite dizzy so I don’t know, it might heal but maybe it would have been good to take a test*.

(Daughter)

The next of kin described a feeling of responsibility to step in and guide nursing staff to ensure that the older person received the appropriate care. They expressed concerns that without their active involvement, important aspects of care may sometimes be overlooked. This included reminding nursing staff about medical follow-ups, ensuring proper medication management, and ensuring that daily routines, such as hygiene and meals, were carried out in a dignified and timely manner.

*They do their job and what they’re supposed to do, but apart from that they don’t take any initiative. ‘Ah now he’s eating badly’ they say to me but go not ahead with it. It feels like it’s up to me to do it*.

(Spouse)

### 3.3. Building Commitment Through Communication and Information

The experiences of the next of kin are important for their continued participation in the older person’s care, highlighting the importance of clear communication and effective information exchange with nursing staff. Communication relies not only on the exchange of information but also on the nursing staff’s commitment to gathering and sharing knowledge about the older person in dialogue with the next of kin.

#### 3.3.1. Dialogue with Nursing Staff

The dialogue with the nursing staff was described in both positive and negative terms. Next of kin expressed a clear wish concerning communication: that nursing staff have a dialogue with them if any changes occur in the older person’s health condition. Many next of kin felt that they often had good dialogue with the nursing staff and that they themselves were seen and listened to. Several of them were actively involved in the care process and initiated regular conversations with the nursing staff. The interactions were seen as crucial for establishing a shared understanding and ensuring the older person’s well-being.

*And as soon as there is something I contact her (the contact person), or the nursing staff contact her and together we discuss. She always calls back and tells me how it has been…*.

(Daughter)

Despite good intentions, some next of kin reported difficulties maintaining dialogue with the nursing staff. Some found it challenging to convey their concerns, leading to frustration and uncertainty. They expressed a desire for their views to be considered even more and to be included by the nursing staff in making decisions regarding the care of their loved ones.

*Yes, instead they can make use of the knowledge that you have as next of kin…. Maybe they’re not used to next of kin interfering. But the only thing I want is for mum to have a good time, they might think I’m a pain, so they’re probably not used to it, I don’t know*.

(Daughter)

They described language difficulties, where their messages were not fully understood, or they received incomplete information about the older person’s condition. The next of kin needed information to know what to, and if they could, contribute to the care.

#### 3.3.2. Remote Communication

Remote communication, primarily through phone calls and text messages, was described as a way for the next of kin to remain informed about the older person’s condition and well-being, particularly when visits were not possible due to distance, work, and family reasons. The frequency of phone calls varied, with some making occasional calls while others maintained daily contact.

*And they (the nursing staff) take pictures and send them to me. We have a cottage by the sea in the summer, we spend a lot of time there, so there is a text message from the nursing staff if she is awake*.

(Daughter)

Those who used remote communication expressed that they valued open and regular communication with nursing staff but were unsatisfied with the timeliness of access and the consistency of updates. It was highlighted that phone calls also served to involve other family members who were unable to visit, ensuring that they remained informed and engaged. Lack of communication was revealed within the organisation, between care levels, as well as a lack of follow-up meetings regarding the care. To facilitate remote communication, initiatives such as placing notebooks in the older person’s room were introduced. These notebooks served as an additional communication tool, allowing both nursing staff and family members to share important information and ensure continuity of care.

Next of kin also stated an underlying frustration and worry that they would not receive a call, when necessary, particularly upon the deterioration of the older person’s health condition, leading to feelings of helplessness and anxiety.

*I was frustrated because I’d come in the afternoon and they’d say, ‘Oh, she’s been so sick since the morning, she’s got a high fever and she’s delirious’. But why didn’t you (the nursing staff) call me before so I could come*?

(Daughter)

This uncertainty reinforced their desire for a more structured and transparent communication system, particularly one that would improve remote communication through different channels to ensure they remained informed about the older person’s condition, especially when they could not be present in person.

#### 3.3.3. Gathering and Sharing Information

The next of kin felt that they played an important role in gathering and sharing information between the older person, nursing staff, other relatives, and relevant authorities. At times, nursing staff showed a strong interest in the older person’s life story, seeing it as a way to maintain their identity. When nursing staff engaged with the person’s past interests, such as baking, and took the opportunity to incorporate these activities into the nursing home, the next of kin felt that the life story was being valued in the care process. In some cases, nursing staff also looked through photo albums with the older person.

*Yes, they play a lot of music and its music that my mum used to listen to when she was young, so… It’s also a way*.

(Daughter)

However, there were other occasions when the next of kin felt that the nursing staff did not show interest in the older person’s past life and experiences, which could have been sustained in a meaningful way. Furthermore, they perceived inconsistencies in information sharing by nursing staff, which sometimes resulted in contradictory or missing updates. These inconsistencies made it difficult for the next of kin to feel assured about the information they received, leading to a sense of insecurity and hindering their ability to stay properly informed. The next of kin stated that they often had to actively request information, as it was not provided continuously. This was due to the frequent replacement of the designated contact person, leaving the next of kin unsure of who to approach. The lack of familiarity with the nursing staff responsible for communication created confusion and made it more difficult for the next of kin to feel informed.

*There is a contact person who is supposed to have the ultimate responsibility, but he or she can’t always be there, and then it’s like everything falls apart. Because not everyone can take responsibility for all the people they care for. If I, as the next of kin, have had contact with a nursing staff, then the person in question has to get in touch with the contact person and give her a report*.

(Daughter)

## 4. Discussion

The study findings reveal the importance of participation in care by the next of kin, achieved through active communication and collaboration with nursing staff at the nursing home. Their involvement in care was an ongoing process that was expressed in the following themes: *Striving to achieve co-created care, Navigating involvement through presence, and Building commitment through communication and information*. Participation was a mutual goal, expressed in dialogue and actions. The staff’s treatment of the next of kin as a resource and the most important person for the older relative ensured that the older person received the best possible care. They balanced being emotionally close to the older relative in their role as next of kin with taking a step back when they were a helping hand in activities that otherwise would be carried out by staff. Drawing boundaries between these dual roles strengthened the relationship with the nursing staff and built trust. Their engagement was multifaceted, ranging from everyday visits to help with daily and personal needs to active advocacy for quality care and older persons’ rights. Their willingness to continue participating in the older person’s care was based on clear communication and effective information exchange with nursing staff. Although the next of kin were able to express what participation meant to them and seek it, not all were allowed to participate. In these situations, they felt frustrated and worried, especially if the older person’s health was deteriorating.

*Striving to achieve co-created care* required constant adjustment. As the older person’s health deteriorated, the responsibilities became more demanding, requiring them to continuously reassess and adapt to new expectations and needs, an often-overwhelming process [8,51]. In Sweden, there is a focus on the patient and next of kin participating in healthcare, which is regulated by law [52,53]. However, the same priority has not been given to municipal care in nursing homes. This is confirmed in an ongoing survey of systematic reviewers by SBU (Swedish Agency for Health Technology Assessment and Assessment of Social Services, unpublished data). This means that healthcare and care services must be planned, implemented, and evaluated in consultation with the recipients of care [52]. It is essential that staff have good interpersonal and communication skills to work preventively when the person they are caring for has an illness or disability that makes it difficult for them to express their wishes and needs. The way staff encounter the patient and their next of kin can have a direct impact on safety when treatment is not person-centred. Misinterpretation of the patient’s symptoms or the problem reported by the next of kin can pose a safety risk, leading to a risky situation. It can also mean that staff do not obtain a clear picture of the person’s symptoms and therefore do not take adequate measures or fail to report symptoms to medically qualified staff [52,54].

In this study, the next of kin did not raise experiences related to Advance Care Planning (ACP). It has been identified internationally as particularly relevant for nursing home residents due to older persons’ multimorbidity and cognitive impairment [9,15]. Despite its recognised importance in palliative care, the literature indicates that only a minority of nursing homes have implemented this tool [16,55], which is in line with the results from this study. ACP can serve as a tool for helping patients communicate their preferences for end-of-life care to the nurse and physician and for next of kin to represent the patient when the older person can no longer communicate their preferences or make decisions on their own [56]. ACP facilitates the involvement of both the older person and their next of kin early in the course of the illness, preparing them for the older person’s impending deterioration [7,55]. Strengthening communication between the next of kin and nursing staff, support structures, and training nursing staff could enhance these processes, ultimately improving the quality of person-centred palliative care [10,14,18].

*Navigating involvement through presence* was illustrated by frequent visits to the nursing homes and a desire to support the older person. Next of kin often found visits particularly challenging when the older person’s health deteriorates, and several experienced feelings of inadequacy and guilt. This pattern aligns with the findings of Rosén et al. [57], who investigated the quality of life of the next of kin of older persons living in nursing homes. The quality of life was affected by feelings of guilt when the older person’s condition deteriorated and their personality changed. A systematic review of qualitative studies on perceptions of good end-of-life care in nursing homes, as reported by the next of kin [58], found that next of kin can experience a crisis when the older person’s condition deteriorates. It is important to involve the next of kin who wish to be present to support shared decision-making. Next of kin often play a central role in care in nursing homes, and previous studies [59,60] have also highlighted the importance of paying attention to and supporting their emotional needs while showing appreciation for their visits to the nursing home.

The significance of *commitment through communication and information in the relationships* between the next of kin and staff is well documented in the literature but has not been highlighted from the perspective of participation in institutional elderly care [61]. Bauer et al. [61] explored the ways in which communication was perceived in the relationship between staff and next of kin: building trust, involvement, and keeping the family happy. Staff attitudes, meaningful engagement, and shared expectations lay the foundations for good relationships [61]. The relationship between staff and the older person, the older person’s emotional well-being, and the provision of person-centred quality care were of paramount importance to the next of kin [61]. Our study found that participation became more complex when there was a sense of time running out as the older person’s health deteriorated, making visits more important but participation in care more difficult. The growing number of next of kin around the world calls for the introduction of policies to give them the right to participate in the care of older people living in nursing homes. In Sweden, support for next of kin caring for an older person at home has been implemented [3]. However, similar self-evident support needs to be developed for institutional care [14].

Although this study shows that participation involves communication and collaboration, few facilitating communication tools were described by the next of kin. Surprisingly, no one mentioned digital communication; the telephone was the most common means of communication for those next of kin who, for whatever reason, could not visit the older person. The placement of a notebook in the older person’s room was also introduced, allowing both nursing staff and family members to share important information and ensure continuity of care.

The next of kin in this study felt reassured when their loved one’s identity and life story were acknowledged and valued by the nursing staff through engagement in the older person’s personal history and past interests. Communication around the older person’s written life story, integrated into daily care, was seen as a key aspect of good collaboration by the next of kin. This finding is in line with Anderssons et al.’s [62] study, which concluded that the next of kin felt that it was important to participate and share the older person’s life stories with nursing staff. This ensures that person-centred care is provided based on the individual’s needs and resources [63] while also enhancing the dignity of an older person living in a nursing home. Implementation of life stories could contribute to a deeper understanding among nursing staff regarding older persons’ family backgrounds, professional lives, and personal interests [64]. In an integrative review of Doran et al. [65], it was highlighted that the use of life stories increased understanding and enhanced nursing staff’s ability to recognise, appreciate, and incorporate life stories into daily care practices. Furthermore, the next of kin felt a sense of comfort when nursing staff actively considered and utilised life stories in their approach to care [64].

However, the findings in this study also found that the next of kin, at times, felt tangible disappointment when nursing staff showed little interest in the older person’s past, and inconsistencies in information sharing led to contradictory or missing updates. Doran and colleagues’ study [65] highlights that life story work benefits from collaboration between nursing staff and the next of kin. Our result aligns with the study by Andersson et al. [62], where the next of kin stated that they sometimes felt uncertain about what information is most relevant to share with nursing staff and how they can best support the creation of a life story. While next of kin are considered a valuable source of information about an older person’s life history, their own time pressures and emotional burden can sometimes limit their ability to contribute effectively [62]. Clear communication and guidance from nursing staff can be essential in facilitating this process. To enhance the use of life stories in elderly care, the next of kin’s unique knowledge and awareness of the older person’s past life should be highlighted in both care planning and daily care [66]. This underlines the importance of capturing the need for recurring nursing staff training, organisational support, clear guidelines, and flexible approaches [65,67]. It can help ensure that life story work is both feasible and beneficial, enriching care practices while respecting the individual needs and preferences of older persons and their next of kin. Our study and previous research suggest that life stories not only support person-centred care but also strengthen relationships between the next of kin, the older person, and the nursing staff.

Since this study is one of four sub-studies in the KUPA project [23,37,49] regarding next of kin’s participation in nursing care, an overview of the main outcomes is presented below, and the results are compared to identify changes following the training.

### 4.1. Discussion of the Results of the Four Studies on the Next of Kin’s Participation

This follow-up study confirms the results of the previous quantitative studies [37,49] by highlighting the core values of participation, i.e., good communication and collaboration. These values were also found in the qualitative study conducted before offering the educational intervention to the nursing staff [23] but were less dominant in the previous interviews with the next of kin compared with this study (Table 5). One possible explanation is that, with time, the next of kin were able to cope with the conflicting feelings of ambivalence. Another explanation is that the educational intervention, consisting of lectures, reflections, and practical training on family involvement in palliative care, had an impact on the extent to which the nursing staff involved the next of kin. The third explanation is that both time and the educational intervention explain the changes. The comparisons of the data show the need for longitudinal studies on participation and that follow-up designs consisting of quantitative and qualitative studies provide valid results. The importance of participation for the quality of life of the next of kin and the older person is another urgent area for future research. The new instrument, the NoK-PiC scale for measuring the participation of next of kin in nursing homes, should also be used in future interventional and longitudinal studies.

### 4.2. Implications of the Results for Future Educational Interventions

The three possible explanations of the improvement of next of kin participation in care after the educational intervention, compared with before, are considered too weak and uncertain. This highlights the need for the development of a palliative care approach to strengthen next of kin participation.

The need for development will be discussed here in line with Froggatt et al. [30]. The three identified levels of implementation of palliative care in nursing homes are macro (national/regional policy, legislation, financial resources), meso (implementation strategies, such as education, tools/frameworks, service models, and research), and micro (palliative care delivery). Their typology was constructed on data from 29 European countries and shows the diversity of implementation activity across Europe. However, it does not include specific activities for the next of kin.

At the macro level, there should be statutory support for the next of kin of people living in nursing homes, similar to the support available in Sweden for those who care for people at home. It is stressful for the next of kin to hand over primary responsibility to staff while watching how their older relative’s health deteriorates [68,69,70,71].

At the meso level, assessing online information systems for next of kin integrated in the educational intervention is a strategy for supporting staff, next of kin, and older relatives. This allows for involvement even by those next of kin who live a long distance from the nursing home. Implementing a digital communication system would keep the next of kin informed of the older person’s condition, especially when they cannot be present in person or when the older person’s condition deteriorates.

At the micro level, there is a need to further test the educational intervention in the KUPA project on other nursing homes not included in this study. Our results highlight the need to include next of kin in the education programme and to establish support groups to strengthen this educational intervention. Online support groups, combined with meetings at the nursing home, can increase dialogue with other next of kin in similar situations, thereby alleviating feelings of guilt and conflicting reactions on their dependence on the staff. The design of the education programme should encourage interaction between next of kin and older people regarding serious decisions that need to be made about palliative care.

### 4.3. Methodological Considerations

Several steps taken throughout this study enhanced its trustworthiness. Participants with different ages, roles (e.g., children, husbands/wives, and siblings), and levels of education were selected to strengthen the transferability of the results to other groups of next of kin of older people. However, only a limited number of men (n = 7) participated in the study. It cannot be ruled out that further variations could have been detected if more men had participated. Another step was site triangulation, as data collection was conducted in 20 nursing homes, both large and small, situated in both urban and rural areas, in different municipalities in two counties. The credibility and transferability of the results were thus ensured due to the inclusion of a range of nursing homes that provided care with different cultures and organisation sizes [72]. Furthermore, the results in this study were also triangulated with results from previous sub-studies on participation. These strengths increase the credibility of the study [72].

Individual interviews were used as the appropriate data collection method since the aim was to explore individuals’ subjective experiences, perceptions, and perspectives on a phenomenon in depth [73]. This method allows participants to reflect on their experiences in a safe environment, where the researcher can ask follow-up questions and deepen their understanding of aspects central to them. Furthermore, interviews enable a nuanced understanding of participants’ thoughts and emotions, which is particularly valuable in studies focusing on experiences within healthcare and social care [74]. The same interviewers as in the study by Wallerstedt et al. [23] applied the same interview guide and basic lines of questioning in this study, increasing the dependability. This data collection method decreased the risk of inconsistency [73].

Several strategies were applied to ensure that the interpretations and descriptions of the findings were embedded in the next of kin’s narratives [75]. To enhance dependability, in line with Sandelowski [76], multiple researchers with different backgrounds were involved in the analysis process and strived to restrain their pre-understanding. The researchers, all skilled and experienced in caring for older persons, were aware of their pre-understanding and strived to maintain a reflexive approach throughout the analysis process. This was achieved through continuous discussions between the researchers, where different perspectives were considered to minimise the risk of prior experiences and assumptions influencing the interpretation of the data. Quotes from the next of kin were used to show that the findings were based on the informants’ own descriptions [72].

This study was conducted in Sweden in the context of nursing homes for older persons. The results from the present study may be applicable to similar contexts; however, it is essential that exploratory studies are conducted to deepen the understanding of a specific target group rather than seeking transferability of the results [72]. When assessing transferability, it is important to be aware of the variation in the international literature surrounding the term ‘nursing home’. To enable meaningful comparison with results from other studies, it is important to clarify the similarities and differences in nursing home practice and nursing home populations [77]. It is also important to take into account the different perspectives on death and palliative care due to ethnicity, religion, and culture. This is highlighted in a systematic scoping review on palliative care for older people, where Sweden was compared with China [78]. Swedish culture is based mainly on Christian traditions, even though Sweden today is relatively secular, in contrast to Chinese culture, which has developed from Taoism, Confucianism, and Buddhism, which have influenced traditional Chinese medicine (TCM) [78]. Studies on international comparisons between different cultures are scarce and are an urgent research area for the future.

Three different researchers conducted the interviews, which may have led to variations in the interview style and the interactions with participants. Despite using the same interview guide, the researchers’ individual approaches to asking questions and interpreting responses could have influenced data collection. To minimise this risk, regular research meetings were held to discuss the interviews, ensuring as much consistency as possible in the methodology. Having multiple interviewers can also be seen as a strength, as it contributes to a broader understanding of participants’ experiences and reduces the risk of a single researcher’s perspective dominating the analysis.

## 5. Conclusions

For next of kin, participation is equivalent to communication and collaboration with nursing staff; this was confirmed by the three other studies on participation in the project. The next of kin had to balance the roles of being responsible for the older person receiving person-centred care while maintaining their own personal space. Over time, the next of kin were more able to cope with conflicting feelings of ambivalence; they experienced quality of life when they knew the older person was receiving good care but struggled with emotional strain when the older person’s condition deteriorated. Acknowledging the older person’s life story was seen as a key element of person-centred care, helping to preserve their dignity. The fear of being ‘a difficult next of kin’ could be interpreted as the communication and collaboration being mostly on the nursing staff’s terms.

Improving the next of kin’s participation requires the development of policy and varied support interventions. Support groups can increase the next of kin’s well-being through dialogue with others in similar situations, thereby alleviating psychological problems such as feelings of guilt, conflicting dependency on staff, and the impending death of the older person. Implementing a digital communication system would keep the next of kin informed of the older person’s condition, especially when they cannot be present in person or when the older person’s condition deteriorates. Further research is needed into how next of kin interact with older persons living in nursing homes, particularly in terms of decision-making regarding palliative care.

## Figures and Tables

**Table 1:** The content of the five seminars published in the study protocol ([36], page 3).

	Themes	Content
Seminar 1	The palliative and dignified approach	The purpose of this theme is to increase knowledge and understanding of the values on which palliative care is based, its ethics, and the approach to dignified patient care.
Seminar 2	Next of kin	The purpose of this theme is to increase knowledge and understanding of the situation and role of the next of kin and to consider how their need for support can be better met.
Seminar 3	Existence and dying	The purpose of this theme is to increase awareness and understanding of one’s own thoughts concerning existence and death with dignity, so that the staff can feel secure partaking in difficult conversations with patients and their next of kin.
Seminar 4	Symptom relief	The purpose of this theme is to increase understanding of the importance of symptom alleviation in palliative care and to promote the use of validated rating instruments.
Seminar 5	Collaborative care	The purpose of this theme is to foster a deeper understanding of the importance of teamwork, with a particular focus on care, which involves collaboration among the staff, the patient, and the next of kin.

**Table 2:** Characteristics of the participating next of kin (n = 36).

	No	%
**Age, years**		
50–59	10	27.7
60–69	17	47.2
70–79	7	19.4
80–89	2	5.5
**Gender**		
Men	7	19.4
Women	29	80.5
**Marital status**		
Married/living together	26	72.2
Unmarried/divorced	7	19.4
Widower/widow	3	
**Relation to the old person**		
Husband/wife	7	19.4
Daughter/son	27	75
Sibling	1	2.7
Other	1	2.7
**Educational level**		
Elementary school	7	19.4
High school	10	27.7
Trade school	3	8.3
University/college	16	44.4
**Work status**		
Full-time	11	30.5
Part-time	7	19.4
Not working	8	22.2
**The frequency of visits to the older person**		
Every day	4	11.1
Weekly	30	83.3
Monthly	1	2.7
Yearly (frequent phone calls)	1	2.7

**Table 3:** Examples of the analytical process.

Data Extracted	Open Coding	Code: Based on Reflections, Interpretation, and Discussion in the Research Group	Sub-Theme	Theme
It would probably have been much easier if I had siblings. Then I might have been able to deal with it differently, knowing that I had family here. But since I am an only child, I have no one. It’s just me and my mother.	If I had siblings, I might have coped differently. But I am an only child, so I have no one.	Role strain	Balancing dual roles and boundaries	Striving to achieve co-created care
…And if the weather is bad, yes, but if it’s raining, then maybe he doesn’t feel like going out. But then, as I’ve said, they can sit and talk to him a little bit instead of going out with him. They don’t have to leave him right away.	If the weather’s bad, he might not want to go out. They can talk to him instead and do not have to leave him.	Encouraging participation	Advocating for quality and rights	Navigating involvement through presence
…Of course, we were contacted by the nurse, and we were in regular contact with her. I continued to visit once a week and sometimes a few extra times. We had discussions with the staff about how she was doing, what had and hadn’t happened, and so on. I think it worked well.	We were in contact with the nurse. I visited once a week and sometimes more. We discussed her progress with staff and it worked well.	Frequent communication	Dialogue with nursing staff	Building commitment through communication and information

**Table 4:** The main findings from interviews with next of kin (n = 37), expressed in themes and sub-themes.

Themes	Sub-Themes
Striving to achieve co-created care	*Balancing dual roles and boundaries*
	*Strengthening the relationship*
	*Building trust*
Navigating involvement through presence	*Visiting the nursing home*
	*A helping hand*
	*Advocating for quality and rights*
Building commitment through communication and information	*Dialogue with nursing staff*
	*Remote communication*
	*Gathering and sharing information*

**Table 5:** The main findings regarding the importance of the next of kin’s participation, expressed as themes from the four sub-studies in the KUPA project.

Publications	Measurement or Themes	Scales, Categories, or Sub-Themes
Westergren et al., 2020 [37]. Measuring next of kin’s experience of participation in the care of older people in nursing homes.	Next of Kin Participation in Care total scale; NoK-PiC scale [37].	Communication and Trust (CaT); Collaboration in Care subscale (CiC).
Westergren et al., 2021 [49]. Next of kin participation in the care of older persons in nursing homes: A pre–post nonrandomised educational evaluation, using within-group and individual person-level comparisons.	Next of Kin Participation in Care total scale; NoK-PiC scale [49].	Communication and Trust (CaT); Collaboration in Care subscale (CiC).
Wallerstedt et al., 2018 [23]. Striking a Balance: A Qualitative Study of Next of Kin Participation in the Care of Older Persons in Nursing Homes in Sweden.	The balancing act between having and leaving responsibility [23].	Visiting the nursing home; Building and maintaining relationships; Gathering and conveying information.
Åvik Persson et al. (the current study). Collaboration and Communication in Care at the Nursing Home: The Next of Kin’s Experiences of Participation After an Educational Intervention to Staff	Striving to achieve co-created care.	Balancing dual roles and boundaries; Strengthening the relationship; Building trust.
	Navigating involvement through presence.	Visiting the nursing home; A helping hand; Advocating for quality and rights.
	Building commitment through communication and information.	Dialogue with nursing staff; Remote communication; Gathering and sharing information.

## Data Availability

The dataset used and analysed during the study is available from the project leader (G.A.) upon written request and in accordance with ethical approval.

## Public Involvement Statement

There was no public involvement in any aspect of this research.

## Guidelines and Standards Statement

This manuscript was drafted according to the COREQ (COnsolidated criteria for REporting Qualitative research) 32-item checklist as a guide.
